# Identification of Serum microRNA-25 as a novel biomarker for pancreatic cancer

**DOI:** 10.1097/MD.0000000000023863

**Published:** 2020-12-24

**Authors:** Yiwen Yu, Ying Tong, Ailing Zhong, Yanchun Wang, Renquan Lu, Lin Guo

**Affiliations:** a Department of Clinical Laboratory, Fudan University Shanghai Cancer Center; b Department of Oncology, Shanghai Medical College, Fudan University, Shanghai, China.

**Keywords:** diagnosis, microRNA, pancreatic cancer, serum

## Abstract

To identify serum microRNA-25 (miR-25) as a diagnostic biomarker for pancreatic cancer (PCa) and to evaluate its supplementary role with serum carbohydrate antigen 19-9 (CA19-9) in early identification of cancers.

Eighty patients with pancreatic cancer and 91 non-cancer controls were enrolled in this study. Quantitative reverse transcription-polymerase chain reaction (RT-PCR) was used to detect the expression level of miR-25. Levels of CA19-9, carcinoembryonic antigen (CEA) and carbohydrate antigen 125 (CA125) were measured by chemiluminescent immunoassay. The logistic model was established to evaluate the correlation of miR-25 with clinical characteristics. A risk model for PCa was conducted by R statistical software. Diagnostic utility for PCa and correlation with clinical characteristics were analyzed.

The expression level of miR-25, in the PCa group was significantly higher (*P* < .05). Risk Model illustrated the relation between miR-25 and pancreatic cancer. With the combination of CA19-9, the performance of miR-25 in early stages (I+II) in the diagnosis of PCa was profoundly better than CA19-9 and miR-25 alone. This combination was more effective for discriminating PCa from non-cancer controls (AUC-ROC, 0.985; sensitivity, 97.50%; specificity, 90.11%) compared with CA19-9 alone or the combination of CA19-9 and CA125.

The expression level of miR-25 among pancreatic cancer patients was significantly higher than that in the control group. miR-25 existed as one of the most relevant factors of PCa. miR-25 can serve as a novel noninvasive approach for PCa diagnosis, and with the supplementary of CA19-9, the combination was more effective, especially in early tumor screening.

## 1 Introduction

Pancreatic cancer (PCa) is one of the most lethal malignancies and aggressive gastrointestinal tumors, remaining the lowest 5-year relative survival rate of 9%. Since its aggressive nature, late pre-diagnosis and limitations of existing chemo and radiation therapies, pancreatic cancer has become the fourth leading cause of cancer types for the estimated death and has been predicted to become the second cause of cancer-related deaths in developed countries by 2030.^[1]^ For 2019, It is estimated that 56,770 new cases will be diagnosed and 45,750 patients will die from it in the USA alone.^[1,2]^ PCa often progresses in absence of its disease-specific signs and symptoms in the early stages, herein, only 20% of cases are surgically resectable at the time of diagnosis, ultimately leading to an extremely poor outcome.

Of all tumor biomarkers, CA19-9, CA125, CEA are those most frequently used for pancreatic cancer detection. Furthermore, it is known to all that CA19-9 ranked the most crucial 1 among 3 biomarkers mentioned above. CA19-9, defined by the monoclonal antibody 1116 NS 19-9, appears as a tumor-related antigen derived from a human colorectal cancer line, reacting with the sialylated Lewis blood group,^[3]^ and consequently plays a vital role in gastrointestinal malignancies, especially in both the diagnosis and the prognosis monitoring of pancreatic cancer.^[4,5]^ In accordance with a pool data analysis of CA19-9 for diagnosing pancreatic cancer, the median sensitivity and specificity was 79% (70%–90%) and 82% (68%–91%), respectively.^[6]^ Whereas, the expression of CA19-9 has also been reported to rise in other conditions, including inflammation, biliary obstruction and other gastrointestinal malignancies.^[5]^ Meanwhile, previous researches have shown that the Lewis (-) individuals which take up the population of approximately 5% to 10%, have minimal or even no secretion of CA19-9, accounting for about 34% of PCa patients.^[7,8]^ In consideration of these conditions, the combination of CA19-9 and CA125 has been widely used in the diagnosis and prognosis of PCa. Herein, there is a pressing need to develop novel and efficient therapeutic biomarkers.

microRNAs (miRNAs) are a class of noncoding, single-stranded 17- to 25-nucleotide-long RNAs, participating in post-transcriptional gene regulation, and impinge indispensable on oncogenesis, tumor metastasis, and diagnosis.^[9]^ miRNAs play such a crucial role in posttranscriptional gene regulation that over 60% of all coding genes are regulated by miRNA as estimated.^[10,11]^ During the past decade, several reports have already verified that dysregulation of miRNA can cause and lead to many cancers.^[12,13]^ Above all, considering the high resistance to endogenous RNase activity as well as temperature changes, miRNAs present highly stable in blood samples, giving them a hallmark to become desirable tumor markers.^[14]^

miR-25 is part of the mir-106b–25 cluster which consists of highly conserved miRNAs: miR-93, miR-106b and miR-25.^[15]^ Several reports have demonstrated the overexpression of miR-25 in numerous types of cancer including gastric cancer,^[16]^ lung cancer,^[17]^ cholangiocarcinoma.^[18]^ Other reports have suggested that miR-25 may act as a tumor suppressor in anaplastic thyroid carcinoma^[19]^ and colon cancer.^[20]^ Recently, Zhang et al have found that the miR-25-3p level is remarkably higher in pancreatic ductal adenocarcinoma (PDAC) than in non-tumor tissues and the overexpression of miR-25-3p can promote both PDAC cell proliferation and metastasis in vivo in mice and in vitro. They put forward that miR-25 in PDAC could be excessively maturated by enhanced N^6^-methyladenosine (m^6^A) modification. PH domain leucine-rich repeat protein phosphatase 2 (PHLPP2) is suppressed by mature miR-25 and miR-25-3p, which leads to the activation of oncogenic AKT-p70S6K signaling, provoking malignant phenotypes of pancreatic cancer cells.^[21]^ Therefore, we tempted to investigate whether miR-25 could become a future biomarker for pancreatic cancer. In this study, we surprisingly found out that the combination of miR-25 and CA19-9 presented a more sensitive diagnostic efficiency which could screen out more pancreatic patients in early stages.

## 2 Materials and methods

### 2.1 Study design and patients

**Figure 1:**
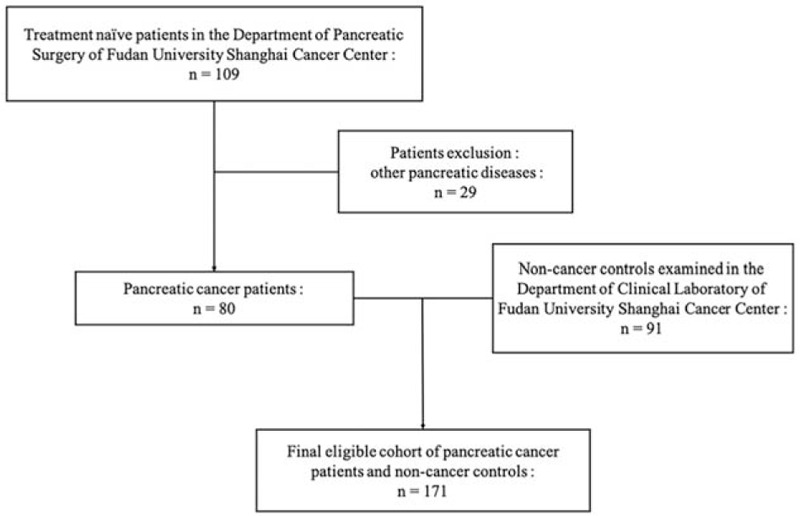
Diagram of study cohort, with exclusion criteria described on the right-hand side of the diagram.

An in-depth analysis of 80 pancreatic cancer patients as well as 91 non-cancer healthy controls were conducted on the basis of a case-control study design. In this study, 80 consecutive patients who had been clinically classified as PCa in Fudan University Shanghai Cancer Center were enrolled finally, from August 2018 to September 2019. The exclusion criteria and study cohort flow diagram are summarized in Figure 1. All blood samples were collected before any therapies, including surgical resection, chemotherapy, and radiotherapy. Any lipemic or hemolyzed blood samples were excluded. Patients were all diagnosed and graded according to the classification of WHO criteria by means of resected tissue specimens, unequivocal histo/cytopathologic evaluation of endoscopic ultrasound-guided fine-needle aspiration Biopsy (EUS-FNAB) and the combination of imaging and biomarkers. Tumors were staged according to the eighth edition of the American Joint Committee on Cancer tumor-node-metastasis (TNM) system. The enrollment criteria were: (1) pathologically confirmed pancreatic adenocarcinoma; (2) no history of other primary malignancies; (3) have not received any treatment. The exclusion criteria were: (1) incomplete clinicopathological data; (2) received chemotherapy or radiotherapy; and (3) acute inflammatory disease, including diseases that can cause secondary diabetes, such as hepatogenic diabetes, Cushing's syndrome, glucagonoma, pheochromocytoma, hyperthyroidism and somatostatin, and other types of diabetes, high blood sugar caused by drugs, etc.

For those patients who have already undergone surgery, definitive tumor stage was established on the basis of operative tissues. For those patients unsuitable for surgical treatment, tumors were staged by means of ultrasonography, magnetic resonance imaging, angiography, dynamic computed tomography, and/or endoscopic ultrasonography.

The non-cancer healthy control population was comprised of 91 normal individuals of matched gender and age without any signs or history of cancer before. Health examination, including serum carcinoembryonic antigen (CEA), carbohydrate antigen 19-9 (CA19-9) and carbohydrate antigen 125 (CA125) and plasma Total Bilirubin (TBIL) test, as well as chest X-ray were conducted to make sure no signs of diseases. Among all 91 normal controls, none of them had been diagnosed with any type of cancer before.

### 2.2 RNA isolation and real-time quantitative PCR (RT-qPCR) assay

Venous blood samples (approximately 5 ml) were collected from each donor and placed in a serum separator tube (BD Biosciences, Basel, Switzerland). Samples were kept under room temperature within 30 minutes and separated by centrifugation at 5000 rpm for 5 minutes at room temperature. Sera in supernatants were recovered and stored at −80°C until RNA extraction.

The RNA simple Total RNA kit (Tiangen, DP419, China) was used along with the manufacturer's instruction to extract serum RNA. Total RNA was extracted from 300 μl of serum by phenol/chloroform purification and centrifugation in isopropyl alcohol. Briefly, 300 μl of serum was mixed with 900 μl of lysate RZ and 240 μl of chloroform. The sample was vortexed and incubated at room temperature for 15 minutes. The mixture was centrifuged at 12,000 g for 10 minutes and the upper aqueous layer was collected. Total RNA (10 μl) was reverse-transcribed into first-strand complementary DNA using a microRNA-25 qualitative detection Kit (MicroMedMark, MM3511, China). RT-qPCR was performed with a microRNA-25 qualitative detection Kit (MicroMedMark, MM3511, China) using an ABI PRISM Detection System (7500, Applied Biosystems, Life Technologies). Amplification curves were generated with an initial denaturing step at 95°C for 5 minutes, followed by 40 cycles of amplification at 95°C for 15 seconds and 60°C for 1 minute. We analyzed the expression levels of the serum miR-25 by absolute quantification. The standard curve is drawn under the concentration of 10^6^, 10^5^, 10^4^,10^3^, with the comparative cycle threshold (Ct) value as the abscissa, and the logarithm of the concentration as the ordinate. The value of regression coefficient r^2^ is greater than 0.99. The amplification efficiency calculated by the slope is between 90% and 110%. RNA concentration is obtained according to the standard curve based on the Ct value of the template. The final relative serum concentration of miR-25 was reached after divided by 6. The normal upper limit is 3333 copies/μl. Since let-7d/g/i in as little as 10 μl of serum have proved to be efficiently detected and reliably compared across multiple samples and remain stable in the serum, the combination was selected as the reference of normalization and the expression levels of miRNAs in this study were normalized to the serum volume directly.^[22]^

### 2.3 Serum CA19-9, CA125, CEA measurement

The concentration of CA19-9, CA125, CEA were all measured by means of chemiluminescent immunoassay (Cobas e601, Loche, Switzerland). The normal upper limit is 27 U/ml, 35 U/ml, and 5.2 ng/ml, respectively. Serum CA19-9, CA125, CEA were measured routinely at baseline.

### 2.4 Statistical analysis

The significance of serum miRNA-25 level was determined by the Mann–Whitney test, Wilcoxon test, the χ^2^ test or Kruskal–Wallis test where appropriate. The reproducibility of RNA extraction and miRNA detection was analyzed by linear correlation. Binary logistic regression analyses were conducted to assess the relationship between miRNA-25 and the incidence of PCa and to find the best logistic model. Receiver operating characteristic (ROC) curves of circulating miRNAs were established for discriminating pancreatic cancer patients from healthy individuals. The area under the ROC curve (AUC) was estimated to evaluate whether miRNA-25 can be a novel predictive marker for PCa. The relationships of miRNAs with clinical characteristics were analyzed by Spearman correlation for categorical items or Pearson correlation for quantitative data. All p-values were two-sided and *P* < .05 was considered statistically significant. All statistical calculations were performed with SPSS version 23.0 (SPSS Inc., Chicago, IL), and the scatter diagram were plotted by GraphPad Prism version 8.0 (GraphPad, San Diego, CA). The pancreatic cancer diagnosis model was established by using R statistical software.

## 3 Results

### 3.1 Patient description

After excluded 29 patients with other pancreatic diseases, all 80 patients enrolled in this study were clinically and pathologically diagnosed with pancreatic cancer (Fig. 1). As shown in Table 1, there were no significant differences in the distribution of age (*P* *=* .096), gender (*P* *=* .170) between cancer patients and non-cancer controls. Whereas, differences in CA19-9, CEA, CA125, TBIL had statistical significances (*P* < .001).

**Table 1:** Characteristics of pancreatic cancer patients and normal controls.

Variable	Controls (n = 91)	Patients (n = 80)	*P* value
Gender (M/F)	36/55	40/40	.170
Age (years, Mean ± SD)	59.98 ± 7.14	62.26 ± 9.51	.096
TNM stage			
I		25	
II		17	
III		21	
IV		17	
CA19-9 (U/ml)			**<.001**^∗^
≤27	89	13	
>27	2	67	
CEA (ng/ml)			**<.001**^∗^
≤5.2	89	54	
>5.2	2	26	
CA125 (U/ml)			**<.001**^∗^
≤35	90	58	
>35	1	22	
TBIL (umol/L)			**<.001**
≤17.1	79	47	
>17.1	12	33	

### 3.2 Detection of a higher level of miR-25 expression in the serum of pancreatic cancer patients than that of non-cancer controls

**Figure 2:**
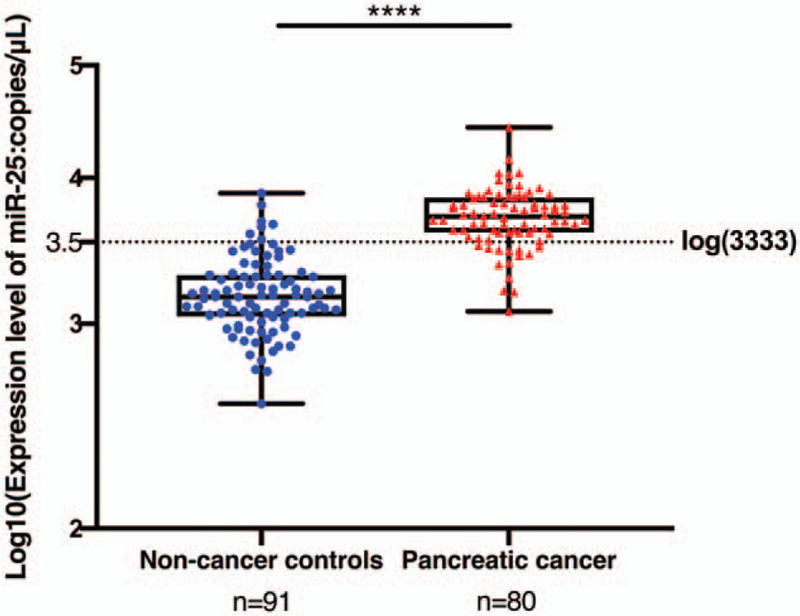
MicroRNA-25 (miR-25) expression levels in the serum of normal controls and pancreatic cancer patients (*P* < .05 by Mann–Whitney *U* test).

We have collected and extracted RNA from sera of pancreatic patients (n = 80) and non-cancer controls (n = 91). Figure 2 shows the expression level of serum miR-25 in both pancreatic cancer patients and controls. Through quantitative RT-PCR, the median absolute dilution values of miR-25 were 5085.75 and 1457.8 in pancreatic cancer patients and normal cases, respectively. Consequently, miRNA array data has revealed a significantly higher expression in the pancreatic cancer group (*P* < .05).

### 3.3 Correlation of serum miR-25 with other common biomarkers for pancreatic cancer

First of all, we conducted a logistic regression model analysis. The results indicated that there existed significant differences among such clinical characteristics as miRNA-25, CA19-9, CEA, CA125 and TBIL in serum after univariate analysis (*P* < .001). There was no significant difference in the results of age and gender after univariate analysis (*P *>* *.05).

After excluding 2 factors, age and gender, which have no significant correlation to pancreatic cancer, remaining pancreatic cancer-related factors were subjected to multivariate logistic regression analysis to screen out other confounding factors. The results revealed that miR-25 and CA19-9 were both independent related factors to pancreatic cancer (*P* < .001). And the OR values of miRNA-25 and CA19-9 were 55.004 and 244.145, respectively, suggesting that miRNA-25 is also closely related to pancreatic cancer and has a potential to become a future biomarker for pancreatic cancer (Table 2).

**Table 2:** Univarate and multivariate analyses for PCa patients using the logistic regressive model.

		Univariate		Multivariate	
	Variables	HR (95% CI)	*P* value	HR (95% CI)	*P* value
Gender	Malefemale	0.655 (0.357-1.201)	0.171		
Age	≥65<65	1.089 (0.574–2.065)	0.794		
miR-25	≥3333<3333	56.571 (21.599–148.171)	**<.001**	55.004 (9.700–311.885)	**<.001**
CA19–9	≥27<27	252.167 (54.610–1164.400)	**<.001**	244.145 (31.075–1918.178)	**<.001**
CEA	≥5.2<5.2	21.426 (4.890–93.881)	**<.001**	7.872 (0.510–121.435)	.139
CA125	≥35<35	34.138 (4.479–260.191)	**.001**	4.542 (0.018–1128.470)	.591
TBIL	≥17.1<17.1	4.622 (2.177–9.814)	**<.001**	2.286 (0.393–13.282)	.357
Constant		0.595	.005		.065

**Figure 3:**
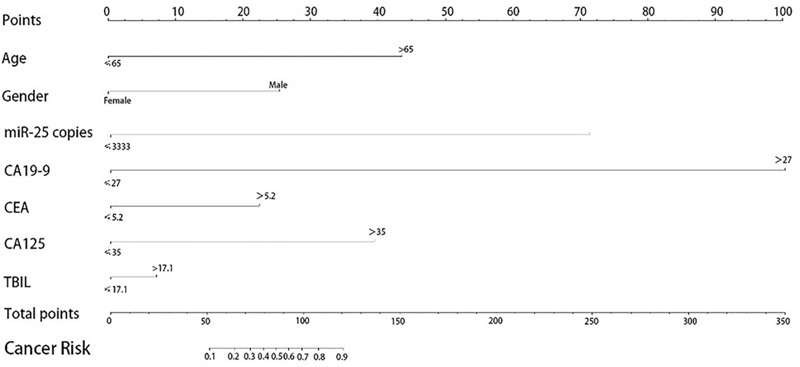
Pancreatic cancer diagnosis model.

Furthermore, in accordance with several clinical characteristics including gender, age, miR-25, CA19-9, CA125, CEA, and TBIL expression levels in the blood and the degree of correlation between pancreatic cancer, we finally established a risk model for pancreatic cancer by nomogram (Fig. 3). As it is shown in Figure 3, the copies of miR-25 and CA19-9 presented more correlated to the risk.

### 3.4 Distribution of miR-25 expression in pancreatic cancer patients and correlation of miR-25

Patient characteristics are all shown in Table 3. Moreover, we analyzed whether there existed a correlation between the serum expression level of miR-25 and clinical characteristics including gender, age, stage, tumor location, T-stage, N-stage, M-stage, CA19-9, CA125, CEA, and TBIL. Among all 80 pancreatic cancer patients, T-stage was the only factor that was relevant to a high level of serum miR-25 (*P* = .031, Table 3).

**Table 3:** Association between serum miR-25 level and clinicopathological characteristics in patients with PCa.

		Serum miR-25	
Variables	n	Median	*P* value
Total	80	5085.75	
Gender			
Female	40	4497.19	.181
Male	40	5545.68	
Ages, years			
≤65	53	5203.74	.733
>65	27	4745.60	
Tumor location			
Phn	40	5429.19	.326
Pbt	40	4497.19	
T-stage			**.031**
T1/T2	45	4510.03	
T3/T4	35	5949.15	
N-stage			.421
N0	40	4781.06	
N1/Nx	40	5107.85	
M-stage			.197
M0	62	4784.04	
M1	18	5810.25	
CEA			.388
<5.2	54	4907.60	
≥5.2	26	5762.85	
CA125			.143
<35	58	4731.36	
≥35	22	5528.50	
TBIL			.147
<17.1	47	4683.75	
≥17.1	33	5232.73	

### 3.5 Tumor stage and diagnostic performance of the combination of miR-25 and CA19-9

Among the PCa group, all the 80 cases were classified by TNM classification from stage I to IV. Pathological diagnosis was the only method to judge a case as positive for PCa. Table 4 mentioned below presents the performance of miR-25, CA19-9, and CA125, which are commonly used in the diagnosis and prognosis of pancreatic cancer in both early (I+II) and advanced (III+IV) stages of pancreatic cancer. The positivity rate for miR-25 at both early and advanced stage was pronouncedly higher than that for CA125 (*P* < .001). This result surprisingly inspired us that the serum expression level of miR-25 can be a novel predictive marker for PCa and furthermore, we proposed a hypothesis whether it could be more efficient and effective than CA19-9 alone for early diagnosis of PCa with the combination of miR-25.

**Table 4:** Performance of miRNA-25, CA19–9, CA125 alone in the differential diagnosis of different stage PCa.

	Stage			
	I+II	III+IV	Total	*P* (Pearson Chi - Squared)
miRNA-25	78.57% (33/42)	86.84% (33/38)	82.50% (66/80)	.331
CA199	73.81% (31/42)	94.74% (36/38)	83.75% (67/80)	**.011**
CA125	9.52% (4/42)	47.37% (18/38)	27.50% (22/80)	**<.001**
*p* value (miR-25 vs CA199)	0.608	0.234^∗^	0.833	
p value (miR-25 vs CA125)	**<0.001**	**<0.001**^∗^	**<0.001**	
miR-25+CA19–9	95.24% (40/42)	100.00% (38/38)	97.50% (78/80)	.558
CA19–9+CA125	73.81% (31/42)	94.74% (36/38)	83.75% (67/80)	**.011**
miR-25+CA125	80.95% (34/42)	89.47% (34/38)	85.00% (68/80)	.286
p value (miR-25+CA19-9 vs CA19-9)	**0.013, 2-sided**	0.495^∗^, 2-sided	**0.005, 2-sided**	
	**0.007, 1-sided**	0.272^∗^, 1-sided	**0.003, 1-sided**	
p value (miR-25+CA19-9 vs CA19-9+CA125)	**0.013, 2-sided**	0.493^∗^, 2-sided	**0.005, 2-sided**	
	**0.007, 1-sided**	0.247^∗^, 1-sided	**0.003, 1-sided**	

Secondly, we analyzed the performance of the combination of CA19-9 and CA125, along with that of CA19-9 and miR-25 in the diagnosis of both early (I+II) and advanced (III+IV) stages of pancreatic cancer. The positivity rate for the combination of miR-25 and CA19-9 in the early stages was pronouncedly higher than that for CA19-9 alone and combination of CA19-9 and CA125 (*P* < .05). These results indicated that serum miR-25 might be a promising predictive marker for PCa and with the combination of CA19-9, it could be significantly effective and efficient for early PCa diagnosis (Table 4).

**Figure 4:**
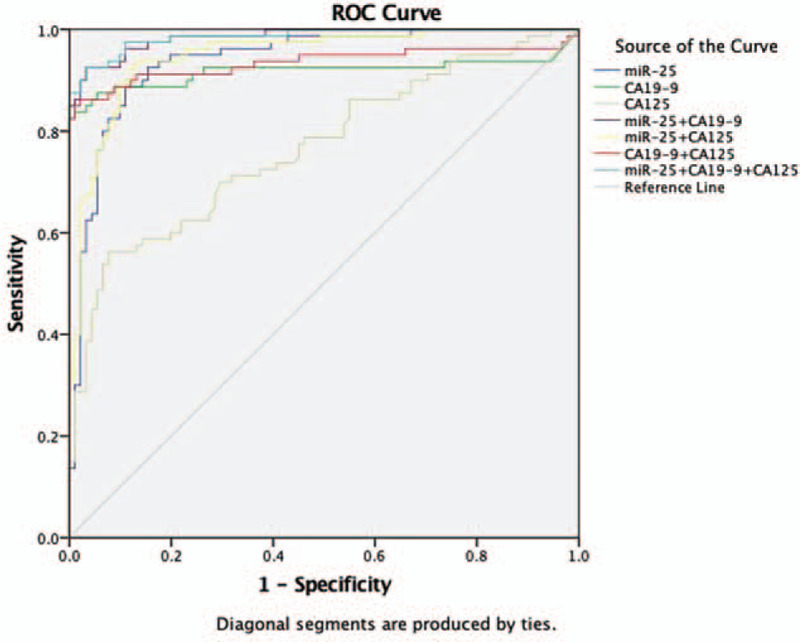
Diagnostic performance of miR-25, CA19-9, CA125, a combination of miR-25 and CA19-9, a combination of CA19-9 and CA125, a combination of miR-25 and CA125 and a combination of miR-25, CA19-9, and CA125. AUC-ROC for detecting PCa from normal (miR-25, 0.939, *P* < .001; CA19-9, 0.918, *P* < .001; miR-25+CA19-9, 0.985, *P* < .001; CA19-9 + CA125, 0.934, *P* < .001; miR-25 + CA125, 0.948, *P* < .001; miR-25 + CA19-9 + CA125, 0.986, *P* < .001).

Thirdly, the ROC curve analysis was performed to evaluate the potential utility of serum miR-25 as a novel biomarker of pancreatic cancer. As it is shown in Figure 4, the sensitivity and specificity of miR-25 for recognizing all pancreatic cancer patients from non-cancer controls were 82.50% and 93.64%, respectively. Meanwhile, the AUC yielded a value of 0.939 (95% CI, 0.903-0.975), which indicated that miR-25 could differentiate PCa patients from normal individuals.

The combination of miR-25 and CA19-9 and that of CA19-9 and CA125 for differentiating PCa from normal folks was assessed by a binary logistic regression model when putting all variables into consideration. All of the 3 variables provided remarkably independent effects.

log itP = −5.842967+0.117874XCA199 + 0.009956X (miR-25/10)
log itP = −3.779 + 0.114XCA199 + 0.047XCA125
log itP = −4.573776 + 0.011581X (miR-25/10) + 0.049573XCA125

The combination of CA19-9 and miR-25 had an AUC-ROC = 0.985 (95% CI: 0.972-0.998), presenting a striking rise compared with CA19-9 alone which yielded an AUC value of 0.918 (95% CI: 0.863-0.973) and the combination of CA19-9 and CA125 with an AUC-ROC = 0.934 (95% CI: 0.888-0.981). These results indicated that this new combination of 2 biomarkers has the potential to become a predictive marker for pancreatic cancer (Fig. 4).

Patients with exact pathological diagnosis was judged as a positive diagnosis for PCa. The sensitivity, specificity, accuracy, positive likelihood ratio (LR+), negative likelihood ratio (LR-), and Youden index of CA19-9 alone, miRNA-25 alone and the combination of both CA19-9 and miR-25 for discriminating PCa are all shown in Table 5. There existed significantly higher diagnostic accuracies of the combination of 2 biomarkers mentioned above, by contrast to both CA19-9 alone and miRNA-25 alone. With regard to the combination of CA19-9 and miR-25, the diagnostic Youden index was >84% (87.61%) with LR-≤0.13 (0.03), which was greatly improved comparing with both CA19-9 alone and the combination of CA19-9 and CA125 (Table 5).

**Table 5:** Performance of miRNA-25, CA19-9, CEA and CA125 and a combination of miRNA-25 and CA19-9 and a combination of CA19-9 and CA125 in the diagnosis of PCa.

	Sensitivity	Specificity	Accuracy	Youden Index	LR+	LR-
miRNA-25	82.50%	93.64%	88.95%	76.14%	12.97	0.19
CA19-9	83.75%	98.18%	92.11%	81.93%	46.02	0.17
CA125	27.50%	98.90%	65.50%	26.40%	25.00	0.73
CA19-9 + CA125	86.25%	96.70%	91.81%	82.95%	26.16	0.14
miRNA-25 + CA19-9	97.50%	90.11%	98.95%	87.61%	9.86	0.03

## 4 Discussion

Pancreatic cancer, with its remarkable mortality and its asymptom in early stage, has already become one of the most dangerous gastrointestinal tumors. Most patients with pancreatic cancer have a poor prognosis due to lymph node infiltration or distant metastasis when diagnosed. At the same time, the cure rate of patients diagnosed at early stage and undergoing surgery is also low. Worse still, half of them died within 2 years. Serum CA19-9 is a tumor-related carbohydrate biomarker, released from a human colorectal cancer cell line which is targeted by the monoclonal antibody 1116-NS-19-9.^[23]^ The concentration of CA19-9 is correlated to the tumor mass, TNM stage, and recurrence.^[3]^ However, it presents poor effectiveness in detecting in the early stages.^[6]^ Therefore, it is urgent to find pancreatic cancer-related biomarkers with higher specificity and sensitivity.

MicroRNAs (miRNAs) are single-stranded, non-coding small fragments of RNA that are found in eukaryotic cells. miRNAs play a role as tumor suppressor genes or oncogenes by regulating genes involved in both tumorigenesis and development at the post-transcriptional level. Of note, miRNAs remain highly stable in the blood, making them potential to become promising tumor markers according to a sort of studies recently, including pancreatic cancer.^[24]^ Over the past few years, studies have reported that miR-20a, miR-21, miR-25, miR-155, miR-196a, and miR-210 are overexpressed in pancreatic cancer tissues and have increased expression levels in patient serum or serum.^[25,26]^

miR-25 emerges as a part of miR-106b–25 gene cluster, which is composed of miR-93, miR-106, and miR-25, and MCM7 is their host gene. As MCM acts as a DNA replication permission factor (RLF), more and more studies have suggested that its expression level is related to tumor invasion ability.^[15]^ Existing reports confirm that miR-106b plays an oncogene role in renal cell carcinoma by affecting cell proliferation, migration, and apoptosis,^[27]^ and miR-25 has been reported in colon cancer, gastric cancer, lung cancer, ovarian cancer, and undifferentiated thyroid cancer.^[16,17,19,28,29]^ At present, there still remains few researches related to miR-25 and pancreatic cancer at home and abroad. To date, Zhang et al has found out that miR-25-3p mutation via m^6^A modification promotes the development and progression of pancreatic cancer,^[21]^ which inspired us to undergo this study.

In this study, RT-qPCR was used to detect the expression level of miR-25 in pancreatic cancer patients and normal controls. The result turned out that the miR-25 concentration in the serum of pancreatic cancer patients was significantly higher than that of non-cancer controls. At the same time, all the samples mentioned above were tested for CA19-9, CEA, CA125, and TBIL, bearing significant differences between PCa group and normal controls. Univariate and multivariate regression modeling was performed by binary classification logistic regression model, and miR-25 and CA19-9, 2 factors most relevant to pancreatic cancer, were screened out. The OR value of miR-25 and CA19-9 is 56.571 and 252.167 (Table 2), respectively, suggesting the correlation with pancreatic cancer. A preliminary risk model for pancreatic cancer was established by using R statistical software. The abnormality of miR-25 and CA19-9 are more relevant with high risk of PCa. Although serum CA19-9 remains the most common diagnostic biomarkers for pancreatic cancer, screening in the early stages of PCa still maintains a challenge. In view of biological functions in tumorigenesis and stability in the blood, it is possible that some cancer-related miRNAs could be future tumor markers, complementary to serum CA19-9 for pancreatic cancer diagnosis and screening in early stages. Our research has surprisingly found out that with the combination of CA19-9 and miR-25, the diagnostic sensitivity in early stages is significantly higher than both CA19-9 alone and the combination of CA19-9 and CA125, which is widely used in the diagnosis and prognosis of PCa. These findings surprisingly indicated this new combination as a novel predictive biomarker for PCa. Unfortunately, we did not find that the level of miR-25 expression was related to the stage and metastasis of pancreatic cancer disease, which may due to the small number of cases. Unlike measuring microRNAs by relative quantification in most recent studies, absolute quantification was used to detect the expression level of miR-25 in serum, making results more accurate and comparable. Further studies are required to expand the sample size.

In summary, this study prompted to develop a more effective serum miRNA with a clinically satisfactory degree of not only the sensitivity but specificity. The level of miR-25 in the serum of patients with PCa is significantly increased. Meanwhile, the elevated level of serum miR-25 is closely correlated with a high risk of PCa. With a combination of CA19-9, this new panel can be a promising predictive marker for PCa, characterized by its high sensitivity and specificity.

## Acknowledgments

We are grateful to the Department of Pancreatic Surgery, Fudan University Shanghai Cancer Center for supporting our research.

## Author contributions

**Conceptualization:** Yiwen Yu, Ying Tong, Yanchun Wang, Lin Guo, Renquan Lu.

**Data curation:** Yiwen Yu, Ailing Zhong, Yanchun Wang.

**Formal analysis:** Yiwen Yu, Ailing Zhong, Yanchun Wang.

**Funding acquisition:** Yanchun Wang, Lin Guo, Renquan Lu.

**Investigation:** Yiwen Yu.

**Methodology:** Yiwen Yu, Ying Tong, Ailing Zhong, Yanchun Wang, Lin Guo, Renquan Lu.

**Project administration:** Lin Guo, Renquan Lu.

**Resources:** Yanchun Wang, Lin Guo, Renquan Lu.

**Supervision:** Lin Guo, Renquan Lu.

**Validation:** Yiwen Yu, Ailing Zhong, Yanchun Wang, Renquan Lu.

**Visualization:** Yiwen Yu, Yanchun Wang.

**Writing – original draft:** Yiwen Yu, Yanchun Wang, Lin Guo, Renquan Lu.

**Writing – review & editing:** Yiwen Yu, Ying Tong, Ailing Zhong, Lin Guo, Renquan Lu.
